# (4-Chloro-3-nitro­benzoato)triphenyl­tin(IV)

**DOI:** 10.1107/S160053681103282X

**Published:** 2011-08-27

**Authors:** Yip-Foo Win, Chen-Shang Choong, Siang-Guan Teoh, Ching Kheng Quah, Hoong-Kun Fun

**Affiliations:** a Department of Chemical Science, Faculty of Science, Universiti Tunku Abdul Rahman, Perak Campus, Jalan Universiti, Bandar Barat, 31900 Kampar, Perak, Malaysia; b School of Chemical Sciences, Universiti Sains Malaysia, 11800 USM, Penang, Malaysia; c X-ray Crystallography Unit, School of Physics, Universiti Sains Malaysia, 11800 USM, Penang, Malaysia

## Abstract

In the title compound, [Sn(C_6_H_5_)_3_(C_7_H_3_ClNO_4_)], the four-coordinate Sn^IV^ atom exists in a distorted tetra­hedral geometry, formed by a monodentate carboxyl­ate group and three phenyl rings. The conformation is stabilized by an intra­molecular C—H⋯O hydrogen bond, which generates an *S*(5) ring. The aromatic ring of the 4-chloro-3-nitro­benzoate ligand makes dihedral angles of 75.64 (12), 64.37 (12) and 2.97 (12)° with the three phenyl ligands. The O atoms of the nitro group are disordered over two sets of sites in a 0.817 (5):0.183 (5) ratio. In the crystal, mol­ecules are linked *via* inter­molecular C—H⋯O hydrogen bonds into chains running parallel to [010].

## Related literature

For general background to and the metal coordination environment of the title complex, see: Win *et al.* (2008 ▶, 2010 ▶, 2011*a* ▶,*b* ▶). For reference bond-length data, see: Allen *et al.* (1987 ▶). For hydrogen-bond motifs, see: Bernstein *et al.* (1995 ▶). For the stability of the temperature controller used in the data collection, see: Cosier & Glazer (1986 ▶).
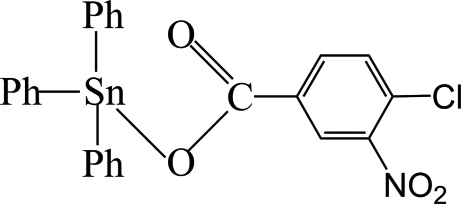


## Experimental

### 

#### Crystal data

[Sn(C_6_H_5_)_3_(C_7_H_3_ClNO_4_)]
*M* *_r_* = 550.54
Monoclinic, 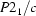

*a* = 12.3926 (2) Å
*b* = 8.8033 (1) Å
*c* = 21.5592 (3) Å
β = 103.217 (1)°
*V* = 2289.72 (6) Å^3^
*Z* = 4
Mo *K*α radiation
μ = 1.26 mm^−1^
*T* = 100 K
0.35 × 0.31 × 0.18 mm

#### Data collection

Bruker SMART APEXII CCD diffractometer
Absorption correction: multi-scan (*SADABS*; Bruker, 2009 ▶) *T*_min_ = 0.668, *T*_max_ = 0.807
23882 measured reflections
5204 independent reflections
4817 reflections with *I* > 2σ(*I*)
*R*_int_ = 0.024

#### Refinement

*R*[*F* ^2^ > 2σ(*F* ^2^)] = 0.028
*wR*(*F* ^2^) = 0.056
*S* = 1.11
5204 reflections
308 parameters
H-atom parameters constrained
Δρ_max_ = 0.60 e Å^−3^
Δρ_min_ = −0.68 e Å^−3^

### 

Data collection: *APEX2* (Bruker, 2009 ▶); cell refinement: *SAINT* (Bruker, 2009 ▶); data reduction: *SAINT*; program(s) used to solve structure: *SHELXTL* (Sheldrick, 2008 ▶); program(s) used to refine structure: *SHELXTL*; molecular graphics: *SHELXTL*; software used to prepare material for publication: *SHELXTL* and *PLATON* (Spek, 2009 ▶).

## Supplementary Material

Crystal structure: contains datablock(s) global, I. DOI: 10.1107/S160053681103282X/hb6362sup1.cif

Structure factors: contains datablock(s) I. DOI: 10.1107/S160053681103282X/hb6362Isup2.hkl

Additional supplementary materials:  crystallographic information; 3D view; checkCIF report

## Figures and Tables

**Table 1:** Selected bond lengths (Å)

Sn1—O1	2.0558 (15)
Sn1—C6	2.121 (2)
Sn1—C18	2.124 (2)
Sn1—C12	2.127 (2)

**Table 2:** Hydrogen-bond geometry (Å, °)

*D*—H⋯*A*	*D*—H	H⋯*A*	*D*⋯*A*	*D*—H⋯*A*
C3—H3*A*⋯O2^i^	0.93	2.55	3.346 (3)	144
C17—H17*A*⋯O1	0.93	2.56	3.140 (3)	120

Symmetry code: (i) 
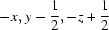
.

**Fig. 1.**
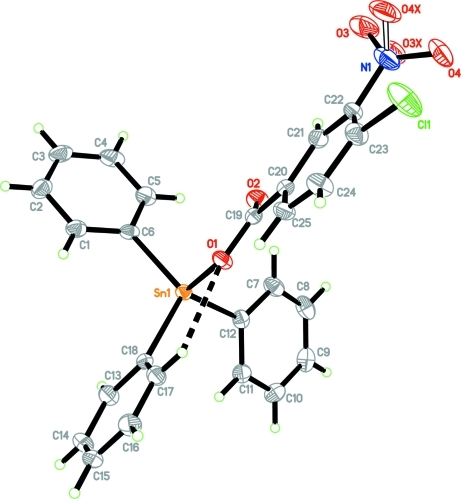
The molecular structure of the title compound showing 50% probability displacement ellipsoids for non-H atoms. Intramolecular hydrogen bonds and minor component of disorder are shown as dashed line and open bonds, respectively.

**Fig. 2.**
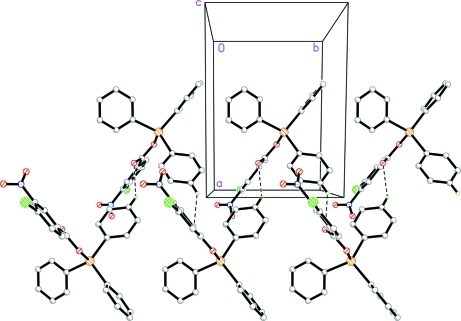
The crystal structure of the title compound, viewed along the c axis. H atoms not involved in hydrogen bonds (dashed lines) have been omitted for clarity. Only the major disorder component is shown.

## supplementary crystallographic information

### Comment

The study of triphenyltin(IV) carboxylate complexes have received tremendous attention due to their structural diversity for which their structure could be monomeric or polymeric although the reaction was carried out in 1:1 molar ratio between the triphenyltin(IV) hydroxide and the respective acid (Win *et al.*, 2008; 2010; 2011*a*,b). In this study, the structure of the title complex is similar to (2-chloro-4-nitrobenzoato)(methanol) triphenyltin(IV) (Win *et al.*, 2011*a*). The only exceptions are that the methanol is not part of the crystal structure and the 2-chloro-4-nitrobenzoic acid is substituted with 4-chloro-3-nitrobenzoic acid.

The molecular structure is shown in Fig. 1. The four-coordinate tin atom (Sn1) exists in a distorted tetrahedral geometry, formed by a monodentate carboxylate group and three phenyl rings. Bond lengths (Allen *et al.*, 1987) and angles are within normal ranges. The molecular structure is stabilized by an intramolecular C17–H17A···O1 hydrogen bond (Table 1), which generates an *S*(5) ring motif (Fig. 1, Bernstein *et al.*, 1995). The phenyl ring (C20-C25) of 4-chloro-3-nitrobenzoate moiety makes dihedral angles of 75.64 (12), 64.37 (12) and 2.97 (12)° with respect to the other three phenyl rings (C1-C6, C7-C12 and C13-C18). Oxygen atoms (O3/O4) of the nitro group are disordered over two positions with refined site-occupancies of 0.817 (5) and 0.183 (5).

In the crystal (Fig. 2), molecules are linked *via* intermolecular C3–H3A···O2 hydrogen bonds (Table 1) into one-dimensional chains parallel to [010] direction.

### Experimental

The title complex was obtained by heating under reflux a 1:1 molar mixture of triphenyltin(IV) hydroxide (1.10 g, 3 mmol) and 4-chloro-3-nitrobenzoic acid (0.60 g, 3 mmol) in methanol (50 ml) for 2 h. A clear transparent solution was isolated by filtration and kept in a bottle. After few days, colourless blocks (1.43 g, 82.1% yield) were collected. Melting point: 408-410 K. Analysis for C_25_H_18_NO_4_ClSn: C, 55.60; H, 3.30; N, 2.55 %. Calculated for C_25_H_18_NO_4_ClSn: C, 54.54; H, 3.29; N, 2.54 %.

### Refinement

All H atoms were positioned geometrically and refined using a riding model with C–H = 0.93 Å and *U*_iso_(H) = 1.2 *U*_eq_(C). Oxygen atoms (O3/O4) of the nitro group are disordered over two positions with refined site-occupancies of 0.817 (5) and 0.183 (5). The highest residual electron density peak and the deepest hole are located at 0.79 and 0.71 Å from atom Sn1, respectively.

### Crystal data

**Table:**

[Sn(C_6_H_5_)_3_(C_7_H_3_ClNO_4_)]	*F*(000) = 1096
*M**_r_* = 550.54	*D*_x_ = 1.597 Mg m^−^^3^
Monoclinic, *P*2_1_/*c*	Mo *K*α radiation, λ = 0.71073 Å
Hall symbol: -P 2ybc	Cell parameters from 9115 reflections
*a* = 12.3926 (2) Å	θ = 2.5–32.7°
*b* = 8.8033 (1) Å	µ = 1.26 mm^−^^1^
*c* = 21.5592 (3) Å	*T* = 100 K
β = 103.217 (1)°	Block, colourless
*V* = 2289.72 (6) Å^3^	0.35 × 0.31 × 0.18 mm
*Z* = 4	

### Data collection

**Table:**

Bruker SMART APEXII CCD diffractometer	5204 independent reflections
Radiation source: fine-focus sealed tube	4817 reflections with *I* > 2σ(*I*)
graphite	*R*_int_ = 0.024
φ and ω scans	θ_max_ = 27.5°, θ_min_ = 1.9°
Absorption correction: multi-scan (*SADABS*; Bruker, 2009)	*h* = −15→16
*T*_min_ = 0.668, *T*_max_ = 0.807	*k* = −11→9
23882 measured reflections	*l* = −28→27

### Refinement

**Table:**

Refinement on *F*^2^	Primary atom site location: structure-invariant direct methods
Least-squares matrix: full	Secondary atom site location: difference Fourier map
*R*[*F*^2^ > 2σ(*F*^2^)] = 0.028	Hydrogen site location: inferred from neighbouring sites
*wR*(*F*^2^) = 0.056	H-atom parameters constrained
*S* = 1.11	*w* = 1/[σ^2^(*F*_o_^2^) + (0.0116*P*)^2^ + 3.5862*P*] where *P* = (*F*_o_^2^ + 2*F*_c_^2^)/3
5204 reflections	(Δ/σ)_max_ = 0.001
308 parameters	Δρ_max_ = 0.60 e Å^−^^3^
0 restraints	Δρ_min_ = −0.68 e Å^−^^3^

### Special details

**Table:**

Experimental. The crystal was placed in the cold stream of an Oxford Cryosystems Cobra open-flow nitrogen cryostat (Cosier & Glazer, 1986) operating at 100.0 (1) K.
Geometry. All esds (except the esd in the dihedral angle between two l.s. planes) are estimated using the full covariance matrix. The cell esds are taken into account individually in the estimation of esds in distances, angles and torsion angles; correlations between esds in cell parameters are only used when they are defined by crystal symmetry. An approximate (isotropic) treatment of cell esds is used for estimating esds involving l.s. planes.
Refinement. Refinement of F^2^ against ALL reflections. The weighted R-factor wR and goodness of fit S are based on F^2^, conventional R-factors R are based on F, with F set to zero for negative F^2^. The threshold expression of F^2^ > 2sigma(F^2^) is used only for calculating R-factors(gt) etc. and is not relevant to the choice of reflections for refinement. R-factors based on F^2^ are statistically about twice as large as those based on F, and R- factors based on ALL data will be even larger.

### Fractional atomic coordinates and isotropic or equivalent isotropic displacement parameters (Å^2^)

**Table:**

	*x*	*y*	*z*	*U*_iso_*/*U*_eq_	Occ. (<1)
Sn1	0.364093 (12)	0.404888 (17)	0.369328 (7)	0.01672 (5)	
Cl1	0.01735 (7)	0.74667 (9)	0.64665 (4)	0.0474 (2)	
O1	0.29596 (13)	0.45731 (19)	0.44511 (8)	0.0217 (3)	
O2	0.18430 (14)	0.6075 (2)	0.37524 (8)	0.0256 (4)	
O3	−0.1368 (2)	0.8214 (4)	0.45615 (14)	0.0435 (8)	0.817 (5)
O4	−0.0736 (2)	0.9462 (3)	0.54411 (12)	0.0375 (7)	0.817 (5)
O3X	−0.0894 (9)	0.9151 (14)	0.4504 (6)	0.038 (3)	0.183 (5)
O4X	−0.1525 (9)	0.8071 (14)	0.5262 (7)	0.049 (4)	0.183 (5)
N1	−0.07089 (19)	0.8404 (3)	0.50478 (13)	0.0344 (5)	
C1	0.22638 (19)	0.1332 (3)	0.30822 (12)	0.0233 (5)	
H1A	0.2741	0.0800	0.3405	0.028*	
C2	0.1424 (2)	0.0565 (3)	0.26581 (12)	0.0293 (6)	
H2A	0.1340	−0.0478	0.2699	0.035*	
C3	0.0713 (2)	0.1352 (3)	0.21756 (13)	0.0326 (6)	
H3A	0.0149	0.0840	0.1895	0.039*	
C4	0.0845 (2)	0.2900 (3)	0.21118 (12)	0.0311 (6)	
H4A	0.0374	0.3426	0.1785	0.037*	
C5	0.1678 (2)	0.3671 (3)	0.25337 (11)	0.0258 (5)	
H5A	0.1761	0.4712	0.2489	0.031*	
C6	0.23910 (18)	0.2891 (3)	0.30242 (11)	0.0200 (5)	
C7	0.3731 (2)	0.7235 (3)	0.30473 (11)	0.0259 (5)	
H7A	0.2964	0.7249	0.2987	0.031*	
C8	0.4268 (2)	0.8430 (3)	0.28291 (12)	0.0293 (6)	
H8A	0.3858	0.9241	0.2621	0.035*	
C9	0.5414 (2)	0.8432 (3)	0.29180 (12)	0.0283 (6)	
H9A	0.5769	0.9245	0.2774	0.034*	
C10	0.6026 (2)	0.7222 (3)	0.32206 (12)	0.0281 (5)	
H10A	0.6793	0.7216	0.3280	0.034*	
C11	0.5490 (2)	0.6014 (3)	0.34361 (11)	0.0234 (5)	
H11A	0.5904	0.5197	0.3636	0.028*	
C12	0.43383 (19)	0.6006 (3)	0.33577 (10)	0.0200 (5)	
C13	0.5609 (2)	0.1802 (3)	0.39217 (12)	0.0249 (5)	
H13A	0.5514	0.1861	0.3482	0.030*	
C14	0.6447 (2)	0.0898 (3)	0.42741 (13)	0.0286 (5)	
H14A	0.6911	0.0358	0.4070	0.034*	
C15	0.6592 (2)	0.0801 (3)	0.49280 (13)	0.0281 (5)	
H15A	0.7153	0.0196	0.5165	0.034*	
C16	0.5902 (2)	0.1608 (3)	0.52298 (12)	0.0275 (5)	
H16A	0.5999	0.1538	0.5670	0.033*	
C17	0.50684 (19)	0.2517 (3)	0.48821 (11)	0.0228 (5)	
H17A	0.4613	0.3061	0.5090	0.027*	
C18	0.49066 (18)	0.2622 (2)	0.42193 (11)	0.0183 (4)	
C19	0.21426 (18)	0.5547 (3)	0.42882 (11)	0.0199 (5)	
C20	0.16128 (17)	0.5965 (3)	0.48230 (11)	0.0188 (4)	
C21	0.07045 (19)	0.6930 (3)	0.47042 (12)	0.0219 (5)	
H21A	0.0401	0.7267	0.4292	0.026*	
C22	0.02544 (19)	0.7387 (3)	0.52057 (12)	0.0230 (5)	
C23	0.0696 (2)	0.6910 (3)	0.58240 (12)	0.0261 (5)	
C24	0.1580 (2)	0.5903 (3)	0.59346 (12)	0.0285 (5)	
H24A	0.1873	0.5546	0.6345	0.034*	
C25	0.20268 (19)	0.5428 (3)	0.54347 (11)	0.0227 (5)	
H25A	0.2612	0.4741	0.5511	0.027*	

### Atomic displacement parameters (Å^2^)

**Table:**

	*U*^11^	*U*^22^	*U*^33^	*U*^12^	*U*^13^	*U*^23^
Sn1	0.01539 (8)	0.01828 (8)	0.01466 (8)	0.00306 (6)	−0.00037 (6)	−0.00178 (6)
Cl1	0.0558 (5)	0.0539 (5)	0.0429 (4)	0.0225 (4)	0.0327 (4)	0.0056 (4)
O1	0.0198 (8)	0.0248 (8)	0.0196 (9)	0.0073 (7)	0.0027 (7)	−0.0023 (7)
O2	0.0247 (8)	0.0332 (10)	0.0166 (9)	0.0060 (8)	−0.0003 (7)	−0.0012 (7)
O3	0.0263 (14)	0.0471 (19)	0.0520 (18)	0.0166 (13)	−0.0017 (13)	0.0013 (14)
O4	0.0418 (14)	0.0267 (13)	0.0495 (16)	0.0145 (10)	0.0218 (12)	0.0025 (11)
O3X	0.029 (6)	0.032 (7)	0.049 (7)	0.016 (5)	0.001 (5)	−0.003 (5)
O4X	0.029 (6)	0.043 (7)	0.085 (10)	0.008 (5)	0.032 (6)	0.005 (6)
N1	0.0292 (12)	0.0284 (12)	0.0495 (16)	0.0116 (10)	0.0168 (12)	0.0074 (11)
C1	0.0188 (11)	0.0301 (13)	0.0201 (12)	0.0018 (10)	0.0028 (9)	−0.0037 (10)
C2	0.0245 (13)	0.0318 (14)	0.0324 (15)	−0.0065 (11)	0.0080 (11)	−0.0114 (11)
C3	0.0187 (12)	0.0471 (17)	0.0305 (15)	−0.0032 (11)	0.0022 (11)	−0.0175 (12)
C4	0.0211 (12)	0.0506 (17)	0.0172 (13)	0.0091 (12)	−0.0047 (10)	−0.0092 (12)
C5	0.0240 (12)	0.0320 (14)	0.0206 (13)	0.0043 (10)	0.0032 (10)	−0.0042 (10)
C6	0.0148 (10)	0.0292 (12)	0.0148 (11)	0.0020 (9)	0.0010 (9)	−0.0059 (10)
C7	0.0270 (12)	0.0304 (13)	0.0201 (12)	0.0085 (11)	0.0050 (10)	0.0011 (10)
C8	0.0425 (15)	0.0243 (12)	0.0230 (13)	0.0103 (11)	0.0111 (12)	0.0061 (10)
C9	0.0423 (15)	0.0237 (12)	0.0209 (13)	−0.0029 (11)	0.0114 (11)	0.0000 (10)
C10	0.0263 (13)	0.0318 (14)	0.0246 (13)	−0.0044 (11)	0.0026 (10)	−0.0015 (11)
C11	0.0254 (12)	0.0237 (12)	0.0182 (12)	0.0018 (10)	−0.0008 (9)	0.0005 (10)
C12	0.0248 (11)	0.0200 (11)	0.0139 (11)	0.0019 (9)	0.0017 (9)	−0.0009 (9)
C13	0.0254 (12)	0.0253 (13)	0.0237 (13)	0.0042 (10)	0.0048 (10)	−0.0006 (10)
C14	0.0231 (12)	0.0262 (12)	0.0360 (15)	0.0079 (10)	0.0059 (11)	−0.0029 (11)
C15	0.0207 (12)	0.0214 (12)	0.0365 (15)	0.0040 (10)	−0.0052 (10)	0.0027 (11)
C16	0.0296 (13)	0.0287 (13)	0.0194 (13)	0.0038 (11)	−0.0043 (10)	0.0013 (10)
C17	0.0221 (11)	0.0219 (12)	0.0232 (13)	0.0039 (9)	0.0026 (10)	−0.0026 (10)
C18	0.0154 (10)	0.0162 (10)	0.0210 (12)	−0.0002 (8)	−0.0007 (9)	−0.0006 (9)
C19	0.0162 (10)	0.0204 (11)	0.0209 (12)	−0.0004 (9)	−0.0003 (9)	−0.0061 (9)
C20	0.0148 (10)	0.0182 (10)	0.0219 (12)	−0.0011 (9)	0.0011 (9)	−0.0038 (9)
C21	0.0202 (11)	0.0200 (11)	0.0229 (13)	0.0020 (9)	−0.0001 (9)	0.0016 (10)
C22	0.0169 (11)	0.0169 (11)	0.0356 (14)	0.0025 (9)	0.0067 (10)	−0.0010 (10)
C23	0.0262 (12)	0.0265 (13)	0.0296 (14)	0.0016 (10)	0.0148 (11)	−0.0003 (10)
C24	0.0276 (12)	0.0350 (14)	0.0235 (13)	0.0080 (11)	0.0071 (10)	0.0028 (11)
C25	0.0172 (11)	0.0257 (12)	0.0244 (13)	0.0052 (9)	0.0032 (9)	−0.0010 (10)

### Geometric parameters (Å, °)

**Table:**

Sn1—O1	2.0558 (15)	C9—C10	1.382 (4)
Sn1—C6	2.121 (2)	C9—H9A	0.9300
Sn1—C18	2.124 (2)	C10—C11	1.389 (3)
Sn1—C12	2.127 (2)	C10—H10A	0.9300
Cl1—C23	1.729 (2)	C11—C12	1.398 (3)
O1—C19	1.311 (3)	C11—H11A	0.9300
O2—C19	1.222 (3)	C13—C14	1.389 (3)
O3—N1	1.185 (3)	C13—C18	1.395 (3)
O4—N1	1.266 (3)	C13—H13A	0.9300
O3X—N1	1.318 (12)	C14—C15	1.383 (4)
O4X—N1	1.239 (10)	C14—H14A	0.9300
N1—C22	1.469 (3)	C15—C16	1.383 (4)
C1—C6	1.390 (3)	C15—H15A	0.9300
C1—C2	1.393 (3)	C16—C17	1.384 (3)
C1—H1A	0.9300	C16—H16A	0.9300
C2—C3	1.385 (4)	C17—C18	1.400 (3)
C2—H2A	0.9300	C17—H17A	0.9300
C3—C4	1.383 (4)	C19—C20	1.497 (3)
C3—H3A	0.9300	C20—C25	1.384 (3)
C4—C5	1.387 (4)	C20—C21	1.387 (3)
C4—H4A	0.9300	C21—C22	1.385 (3)
C5—C6	1.394 (3)	C21—H21A	0.9300
C5—H5A	0.9300	C22—C23	1.385 (4)
C7—C8	1.384 (4)	C23—C24	1.387 (3)
C7—C12	1.398 (3)	C24—C25	1.384 (3)
C7—H7A	0.9300	C24—H24A	0.9300
C8—C9	1.388 (4)	C25—H25A	0.9300
C8—H8A	0.9300		
			
O1—Sn1—C6	106.24 (7)	C11—C10—H10A	120.2
O1—Sn1—C18	95.50 (7)	C10—C11—C12	121.1 (2)
C6—Sn1—C18	114.46 (9)	C10—C11—H11A	119.4
O1—Sn1—C12	111.17 (8)	C12—C11—H11A	119.4
C6—Sn1—C12	116.64 (9)	C7—C12—C11	118.5 (2)
C18—Sn1—C12	110.66 (9)	C7—C12—Sn1	125.01 (18)
C19—O1—Sn1	111.47 (14)	C11—C12—Sn1	116.48 (17)
O3—N1—O4X	80.8 (7)	C14—C13—C18	120.8 (2)
O3—N1—O4	125.3 (3)	C14—C13—H13A	119.6
O4X—N1—O4	77.0 (6)	C18—C13—H13A	119.6
O3—N1—O3X	48.6 (5)	C15—C14—C13	120.0 (2)
O4X—N1—O3X	117.3 (8)	C15—C14—H14A	120.0
O4—N1—O3X	101.7 (5)	C13—C14—H14A	120.0
O3—N1—C22	118.6 (2)	C14—C15—C16	119.9 (2)
O4X—N1—C22	117.3 (6)	C14—C15—H15A	120.1
O4—N1—C22	116.0 (2)	C16—C15—H15A	120.1
O3X—N1—C22	118.5 (5)	C15—C16—C17	120.5 (2)
C6—C1—C2	120.2 (2)	C15—C16—H16A	119.8
C6—C1—H1A	119.9	C17—C16—H16A	119.8
C2—C1—H1A	119.9	C16—C17—C18	120.4 (2)
C3—C2—C1	120.1 (3)	C16—C17—H17A	119.8
C3—C2—H2A	119.9	C18—C17—H17A	119.8
C1—C2—H2A	119.9	C13—C18—C17	118.5 (2)
C4—C3—C2	119.9 (2)	C13—C18—Sn1	121.51 (17)
C4—C3—H3A	120.1	C17—C18—Sn1	120.00 (16)
C2—C3—H3A	120.1	O2—C19—O1	123.5 (2)
C3—C4—C5	120.3 (3)	O2—C19—C20	122.8 (2)
C3—C4—H4A	119.9	O1—C19—C20	113.7 (2)
C5—C4—H4A	119.9	C25—C20—C21	119.6 (2)
C4—C5—C6	120.2 (3)	C25—C20—C19	121.2 (2)
C4—C5—H5A	119.9	C21—C20—C19	119.3 (2)
C6—C5—H5A	119.9	C22—C21—C20	119.2 (2)
C1—C6—C5	119.3 (2)	C22—C21—H21A	120.4
C1—C6—Sn1	119.58 (18)	C20—C21—H21A	120.4
C5—C6—Sn1	121.11 (18)	C21—C22—C23	121.5 (2)
C8—C7—C12	120.2 (2)	C21—C22—N1	116.6 (2)
C8—C7—H7A	119.9	C23—C22—N1	121.9 (2)
C12—C7—H7A	119.9	C22—C23—C24	118.8 (2)
C7—C8—C9	120.6 (2)	C22—C23—Cl1	123.18 (19)
C7—C8—H8A	119.7	C24—C23—Cl1	118.0 (2)
C9—C8—H8A	119.7	C25—C24—C23	120.0 (2)
C10—C9—C8	119.9 (2)	C25—C24—H24A	120.0
C10—C9—H9A	120.1	C23—C24—H24A	120.0
C8—C9—H9A	120.1	C24—C25—C20	120.8 (2)
C9—C10—C11	119.7 (2)	C24—C25—H25A	119.6
C9—C10—H10A	120.2	C20—C25—H25A	119.6
			
C6—Sn1—O1—C19	−65.58 (16)	C14—C13—C18—Sn1	178.65 (19)
C18—Sn1—O1—C19	176.95 (15)	C16—C17—C18—C13	−0.6 (4)
C12—Sn1—O1—C19	62.28 (16)	C16—C17—C18—Sn1	−179.01 (18)
C6—C1—C2—C3	0.2 (3)	O1—Sn1—C18—C13	174.44 (19)
C1—C2—C3—C4	0.6 (4)	C6—Sn1—C18—C13	63.8 (2)
C2—C3—C4—C5	−0.8 (4)	C12—Sn1—C18—C13	−70.5 (2)
C3—C4—C5—C6	0.2 (4)	O1—Sn1—C18—C17	−7.14 (19)
C2—C1—C6—C5	−0.7 (3)	C6—Sn1—C18—C17	−117.78 (18)
C2—C1—C6—Sn1	177.96 (16)	C12—Sn1—C18—C17	107.94 (19)
C4—C5—C6—C1	0.5 (3)	Sn1—O1—C19—O2	−0.7 (3)
C4—C5—C6—Sn1	−178.13 (17)	Sn1—O1—C19—C20	179.81 (14)
O1—Sn1—C6—C1	−85.63 (18)	O2—C19—C20—C25	−175.2 (2)
C18—Sn1—C6—C1	18.4 (2)	O1—C19—C20—C25	4.3 (3)
C12—Sn1—C6—C1	149.83 (17)	O2—C19—C20—C21	3.5 (3)
O1—Sn1—C6—C5	93.03 (18)	O1—C19—C20—C21	−177.0 (2)
C18—Sn1—C6—C5	−162.96 (17)	C25—C20—C21—C22	2.5 (3)
C12—Sn1—C6—C5	−31.5 (2)	C19—C20—C21—C22	−176.3 (2)
C12—C7—C8—C9	−0.4 (4)	C20—C21—C22—C23	0.5 (4)
C7—C8—C9—C10	0.7 (4)	C20—C21—C22—N1	−179.7 (2)
C8—C9—C10—C11	−0.2 (4)	O3—N1—C22—C21	37.1 (4)
C9—C10—C11—C12	−0.6 (4)	O4X—N1—C22—C21	131.5 (8)
C8—C7—C12—C11	−0.5 (3)	O4—N1—C22—C21	−140.2 (2)
C8—C7—C12—Sn1	−177.80 (18)	O3X—N1—C22—C21	−18.8 (7)
C10—C11—C12—C7	1.0 (3)	O3—N1—C22—C23	−143.2 (3)
C10—C11—C12—Sn1	178.53 (18)	O4X—N1—C22—C23	−48.8 (8)
O1—Sn1—C12—C7	−69.0 (2)	O4—N1—C22—C23	39.5 (3)
C6—Sn1—C12—C7	53.0 (2)	O3X—N1—C22—C23	160.9 (7)
C18—Sn1—C12—C7	−173.79 (19)	C21—C22—C23—C24	−2.8 (4)
O1—Sn1—C12—C11	113.67 (17)	N1—C22—C23—C24	177.5 (2)
C6—Sn1—C12—C11	−124.34 (17)	C21—C22—C23—Cl1	179.23 (19)
C18—Sn1—C12—C11	8.8 (2)	N1—C22—C23—Cl1	−0.5 (4)
C18—C13—C14—C15	0.1 (4)	C22—C23—C24—C25	2.0 (4)
C13—C14—C15—C16	−0.1 (4)	Cl1—C23—C24—C25	−179.9 (2)
C14—C15—C16—C17	−0.2 (4)	C23—C24—C25—C20	1.0 (4)
C15—C16—C17—C18	0.6 (4)	C21—C20—C25—C24	−3.2 (4)
C14—C13—C18—C17	0.2 (4)	C19—C20—C25—C24	175.5 (2)

### Hydrogen-bond geometry (Å, °)

**Table:**

*D*—H···*A*	*D*—H	H···*A*	*D*···*A*	*D*—H···*A*
C3—H3A···O2^i^	0.93	2.55	3.346 (3)	144
C17—H17A···O1	0.93	2.56	3.140 (3)	120

Symmetry codes: (i) −*x*, *y*−1/2, −*z*+1/2.
